# Determinants of pain, functional limitations and health-related quality of life six months after total knee arthroplasty: results from a prospective cohort study

**DOI:** 10.1186/2052-1847-5-2

**Published:** 2013-03-28

**Authors:** François Desmeules, Clermont E Dionne, Étienne L Belzile, Renée Bourbonnais, François Champagne, Pierre Frémont

**Affiliations:** 1 School of Rehabilitation, Faculty of Medicine, Université de Montréal, CP 6128 Succursale Centre-Ville, H3C 3J7, Montréal, QC, Canada; 2 URESP du Centre de recherche FRSQ du CHA Universitaire de Québec, Quebec City, QC, Canada; 3 University of Montreal Public Health Research Institute, Université de Montréal, Montreal, QC, Canada; 4 Department of Rehabilitation, Faculty of Medicine, Université Laval, Quebec City, QC, Canada; 5 Laval University Hospital Center (CHUQ), Quebec City, QC, Canada; 6 Community Health Care Centre (CSSS) de la Vieille-Capitale, Quebec City, QC, Canada; 7 Laval University Hospital Research Center (CRCHUQ), Quebec City, QC, Canada

**Keywords:** Determinants, Disability, Health related quality of life (HRQoL), Osteoarthritis and Total knee arthroplasty

## Abstract

**Background:**

Total knee arthroplasty (TKA) is an effective procedure. However, for some patients, the outcomes are not satisfactory. Identification of TKA determinants could help manage these patients more efficiently. The purpose of this study was to identify pre- and perioperative determinants of pain, functional limitations and health-related quality of life (HRQoL) 6 months after TKA.

**Methods:**

138 participants were recruited from 3 hospitals in Quebec City, Canada and followed up until 6 months after surgery. Data were collected through review of the subjects’ medical files and structured telephone interviews before and 6 months after TKA. Pain and functional limitations were measured with the Western Ontario and McMaster Osteoarthritis Index (WOMAC) and HRQoL was measured with the SF-36 Health Survey. Independent variables included demographic, socioeconomic, psychosocial, clinical and surgical characteristics of participants as well as data on health services utilization. Stepwise multiple regression analysis was used to assess the strength of the associations between the independent variables and the WOMAC and SF-36 scores.

**Results:**

Higher preoperative pain, cruciate retaining implants and the number of complications were significantly associated with worse pain 6 months after TKA (p < 0.05) and explained 11% of the variance of the WOMAC pain score. Higher preoperative functional limitations, being single, separated, divorced or widowed, being unemployed or retired and the number of complications were significantly associated (p < 0.05) with worse functional limitations 6 months after TKA and explained 16% of the variance of the WOMAC function score. Lower preoperative HRQoL, contralateral knee pain, higher psychological distress and comorbidities were significantly associated (p < 0.05) with worse HRQoL 6 months after TKA and explained 23% of the variance of the SF-36 physical functioning score.

**Conclusions:**

Several variables were found to be significantly associated with worse outcomes 6 months after TKA and may help identify patients at risk of poorer outcome. The identification of these determinants could help manage patients more efficiently and may help target patients who may benefit from extensive rehabilitation.

## Background

Total knee arthroplasty (TKA) is a common surgical procedure that allows for an effective reduction of pain and adequate restoration of function for the majority of patients suffering from advanced knee osteoarthritis or other forms of arthritis [1,2]. There has been an escalating demand for TKA in the past decade, making it the second most popular orthopaedic surgery. Predictions suggest this trend will continue for years to come and access to TKA will still be an issue in many states, provinces and territories across North America [3,4]. Despite the addition of health care resources to address this problem, new strategies to improve management of individuals undergoing TKA and optimize surgical outcomes are needed to avert the rising demand and efficiently allocate resources to this clientele.

Although TKA is an efficacious intervention for treating pain, loss of function and diminished HRQoL, 10 to 30% of patients report poor outcomes or no improvement following the intervention [5-8]. Identifying patients who are at risk of complications or may encounter a poorer outcome following surgery is therefore an important issue. Identifying such patients could help clinicians and patients themselves in making the decision to go forward with such an intervention [9] or it may lead to the implementation of medical or rehabilitation interventions to help these patients before surgery and after surgery. For example, patients identified as potentially at risk of a poorer outcome before surgery, could be enrolled in a prehabilitation program or intensive rehabilitation could be planned following surgery [10]. Ultimately, in terms of health service organization, identification of patients at risk of poorer outcomes following TKA may also allow stakeholders and clinicians to better plan healthcare resources needed for theses patients [2,10].

The identification of factors affecting the outcomes of TKA and of patients at risk of poorer outcomes remains a challenge. The outcomes of TKA are clearly complex and investigations of possible determinants have been primarily directed toward perioperative surgical complications and prosthetic-related factors [2]. Surgical factors such as prosthesis design, implant type (cruciate retaining or postero-stabilized), bearing type (mobile or fixed), may play a small role in the short term outcomes but ultimately seemed to have more of an impact on the longevity of the prosthesis. Perioperative or surgical complications, although their frequency is low, may play a more important role in the short-term as well as the long-term outcomes of TKA, depending on the nature and severity of the complications [2,9]. Preoperative status is one of the few factors that has been consistently associated with post-operative status in terms of pain, function and HRQoL following total joint arthroplasty [10-16]. Many other personal, clinical or psychosocial factors have been associated with worse pain, function and HRQoL following TKA, however results have not been consistent across studies. These factors include: older age, female gender, low income, low formal education, high body mass index (BMI), longer disease duration, comorbidities, pre-operative use of a walking aid, depressive symptoms and low social support, but again, theses findings are not consistent and the precise impact or strength of the association between these factors and the outcomes remain elusive [6,9-11,13-20].

It thus remains a challenge to identify which TKA candidates will likely do well, or do poorly following TKA and may need extensive rehabilitation [2,9]. The purpose of the current study was therefore to identify, in patient undergoing TKA, preoperative and perioperative determinants of pain, functional limitations and health related quality of life (HRQoL) 6 months after surgery.

## Methods

### Study design

This study adopted a longitudinal prospective design with repeated measures. It was part of a broader investigation that measured the impacts of pre-surgery wait in patients undergoing TKA [21,22].

### Settings

From 02/2006 to 09/2007, patients were recruited from the surgical wait lists of the departments of orthopaedic surgery of 3 university hospitals in Quebec City, Canada (CHUL, HSFA and HDQ). Because of the extensive wait times, the post-surgery follow-up was only concluded in 2010. All 7 surgeons performing elective TKA in these hospitals participated in the study.

### Participants

Every week, patients newly enrolled on the wait lists of the hospitals were contacted by a research nurse. Eligible subjects had to meet the following inclusion criteria: 1- Aged ≥40 years; 2- Contacted within 3 weeks of being enrolled on the orthopaedic wait lists for primary unilateral TKA; 3- Resident of the province of Quebec, with provincial universal health insurance coverage; 4- Understand and speak French. Patients were excluded if they were suffering from a severe cardiac condition, any severe degenerative disease (except osteoarthritis) such as Alzheimer's disease, Parkinson's disease, any type of dystrophies, multiple sclerosis or other type of sclerosis that could interfere with the patient recovery following surgery or if they were suffering from a severe mental disorder that could interfere with the ability to answer the protocol questionnaires (severe depression, bipolar disorders, dementia or schizophrenia). Patients with a previous joint arthroplasty (hip or knee) were also excluded. Those who suffered a major trauma to the knee in the previous year or underwent surgery urgently within 30 days of being assigned on the wait list were further excluded.

### Data collection

Data were collected through review of the subjects’ medical files and structured 45 minutes telephone interviews conducted by trained interviewers. The interviews were conducted a few days before surgery (mean ± SD: -5.7 ± 3.4 days) and 6 months after TKA (mean ± SD: 188.7 ± 5.4 days). The results of another interview that took place at the inclusion on the surgical wait list, regarding the effects of wait time, have been reported previously [22,23].

#### Dependent variables

The first dependent variable was the Western Ontario and McMaster Osteoarthritis Index (WOMAC), which measures pain and functional limitations related to the knee [24]. WOMAC scores were transformed in order to obtain a range from 0 to 100, where a score of 100 indicated no pain or any functional limitations. The French-Canadian 5 point likert version was used [25]. The WOMAC has been found to have very good reliability, convergent construct validity and responsiveness, and has been used extensively in similar populations and is suitable for telephone administration [25-28].

The second dependent variable addressed HRQoL and was measured with the Medical Outcomes Study 36-Item Short Form Health Survey (SF-36), a generic questionnaire on health status and HRQoL related to 8 dimensions of health [29]. It allows for the calculation of a specific scale for each of the 8 health dimensions. The score ranges from 0 to 100, where 100 indicates optimal HRQoL. The French-Canadian version was used. Use of the SF-36 has been extensive in this population [6,10,19,29-32]. The reliability and validity of this questionnaire have been well established [33-35]. Only the 3 more responsive health domain scores related to physical health (physical functioning, role-physical and bodily pain) are presented in this paper [36,37].

#### Independent variables

Marital status, household living status (living alone or not), and clinical variables such as initial diagnosis (osteoarthritis or rheumatoid arthritis), body mass index (BMI) and comorbidities were collected through review of the subjects’ medical charts. The Cumulative Illness Rating Scale (CIRS) was used to assess the burden of comorbidities. This index measures the burden of chronic illness while taking into consideration the severity of chronic diseases through the review of 14 body systems. Each system is scored on a scale of 0 to 4 where 0 = no problem and 4 = extremely severe problem. The total score ranges from 0 to 56 and was kept as a continuous variable for the statistical analyses. This tool has been found to be reliable and valid in various settings [38-40]. The CIRS was scored by one of the investigator (PF), a sports medicine physician.

Duration of disease symptoms (years) and use of a walking aid were also documented. Pain in the contralateral knee was assessed using the 5 questions of the WOMAC pain scale. For the analyses, the score was dichotomized (presence or absence of contralateral knee pain). Formal education, employment status, household income and social support were measured with questions drawn from the questionnaire of the 1998 Quebec Health Survey [41]. The validated and reliable social support measurement tool has 3 sections that refer to the size of the social network, satisfaction with social life and social integration [42]. Because of time constraints, only the questions regarding the size of the social network were used in this research. For analyses, the social support score (range: 0–150) was dichotomized around the median score.

Psychological distress was documented with a modified version of the Psychological Symptom Index (PSI), that measures depression and anxiety during the last week (range: 0–42) [43]. Its French adaptation has been performed by Préville et al. (1992) and has been found highly reliable [44,45]. For analyses the PSI score was kept as a continuous variable.

Surgical variables such as type of implant, bearing type, implant fixation, patella resurfacing and the number and type of in-hospital complications (wound infection, implant infection, fracture or dislocation, knee ankylosis and manipulation, cardiovascular/pulmonary/circulatory complications, peripheral/central nervous system involvement, urinary infection, acute confusion, tendon and ligament rupture, blood transfusion) following TKA were documented through the review of the subjects’ medical files [19]. Hospital length of stay and discharge to a rehabilitation or recovery facility were also documented through chart review.

Six months after surgery, patients were asked about the number of community physiotherapy treatment hours they received following discharge from the hospital.

### Analyses

Descriptive statistics were used to summarize subjects’ characteristics as well as WOMAC and SF-36 scores before and after surgery. Less than 2% of the data on the WOMAC and SF-36 questionnaires were missing. Missing data was handled according to both tools respective guidelines [24,29]. Paired Student t-tests and ninety-five percent confidence intervals (95% CI) were used to assess overall change in the mean WOMAC and SF-36 scores from before surgery to 6 months after TKA.

Stepwise multiple regression analysis was used to assess the strength of the associations of the independent variables considered with the WOMAC and SF-36 scores. Significance levels for independent variable selection were set at 0.10 for initial model entry and at 0.05 to remain in the final model. Confounding was defined as a change ≥ 10% in the regression coefficient of at least one independent variable of a model [46]. When dependent variables showed non-normal distributions, the scores were transformed into ranks [47]. Residual plots, outliers and multicollinearity of final models were also assessed. Assuming a type I error (α) of 0.05, power (1-β) of 0.80 and including up to 5 independent variables in the final models, a sample size of 61 subjects would be needed to detect an explained variance of at least 19% on the WOMAC function scale(r = 0.43) and a sample size of 110 subjects would be needed to detect an explained variance of at least 11% on the WOMAC function scale (r = 0.33) [10,13,48]. A 19% change in the WOMAC score and an 11% change on the SF-36 is considered a clinically important difference in a population undergoing TKA [49]. Statistical analyses were performed with the SAS software version 9.2 for Windows (SAS Institute Inc, Cary, NC, USA).

### Ethics

All participants signed an informed consent form. The study was approved annually by the Research Ethics Boards of all 3 hospitals.

## Results

### Participants

**Figure 1:**
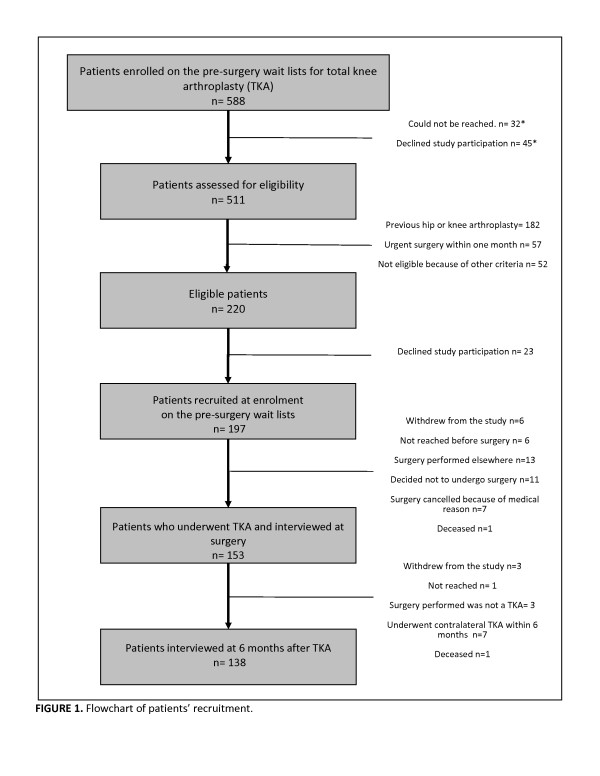
**Flowchart of patients’ recruitment.** This study was part of a broader investigation that measured the impacts of pre-surgery wait in patients undergoing TKA and recruitment was therefore done at the enrollment on the pre-surgery wait list and patient were followed during pre-surgery wait. Specific to the objectives of the current paper, of the 153 participants who completed the interview conducted just before surgery, 138 answered the interview conducted 6 months after TKA. *Eligibility status unknown. Considered in initial participation proportion calculation.

Figure 1 presents the flow of patients considered and recruited for the entire study. Overall, 588 consecutive patients were enrolled on the pre-surgery wait lists during the recruitment period. 220 patients were found eligible, of whom 197 accepted to participate. 45 patients refused to participate before eligibility was assessed and 32 could not be contacted within 3 weeks. The 32 patients who could not be contacted were included in the calculation of the overall eligibility proportion [(220 + 32) / (588–45) = 0.464]. Calculation of the initial participation proportion included the eligible patients and the 45 patients who refused to participate before eligibility assessment [197 / (220 + (45 × 0.464)) = 81.8%].

Specific to the objectives of the current paper, of the 153 participants who completed the interview conducted just before surgery, 138 answered the interview conducted 6 months after TKA. Three had a hemiarthroplasty performed instead of a TKA, 7 participants underwent contralateral TKA within 6 months of the first TKA and 1 patient died (unrelated cause). Three participants withdrew from the study and one could not be reached (follow-up proportion: 138 /153 = 90,1%).

### Participants’ characteristics and overall changes in WOMAC and SF-36 scores

Table 1 presents selected characteristics of the participants of this study and Table 2 present overall changes from before surgery to 6 months after TKA.

**Table 1:** Selected characteristics of the study participants who underwent primary unilateral total knee arthroplasty (n = 138)

**Variables**	**n (%)**	**Mean (SD)**	**Variables**	**n (%)**	**Mean (SD)**
**Demographics**			**Clinical characteristics**		
** Age** (years)		67 (9.3)	** Diagnosis**		
			Osteoarthritis	133 (96)	
** Gender**			Rheumatoid arthritis	5 (4)	
Female	91 (66)		** BMI **^‡^ (kg/m^2^)		31.2 (6.2)
** Marital status**			** Comorbidities** ( /56)		6.5 (2.2)
Single, separated, divorced or widowed	51 (37)		** Duration of knee symptoms before enrolment (years) **^§^ (years)		8.2 (8.3)
Married or common law	87 (63)		** Contralateral knee pain **^∣∣^	100 (72)	
			** Use of a walking aid before surgery**	52 (38)	
** Living alone**	33 (24)		**Surgical characteristics**		
			** Implant type**		
**Socioeconomic characteristics**			Postero-stabilized	108 (78)	
** Educational level** (part or complete)			Cruciate retaining	30 (22)	
High school or less	77 (56)		** Implant fixation**		
College or university	61 (44)		Cementless	4 (3)	
** Employment status**			Hybrid	2 (1)	
Unemployed or retired	108 (78)		Cemented	132 (96)	
Employed	30 (22)		** Implant bearing type**		
Household income ^*^			Mobile	4 (3)	
< $30 000 / year	48 (35)		Fixed	134 (97)	
$30 000 - $59 999/ year	42 (30)		** Patella resurfacing**	129 (93)	
≥ $60 000/ year	32 (23)		** Number of in-hospital complications**^¶^		
			0	103 (77)	
**Psychosocial characteristics**			1	34 (24)	
** Psychological distress** (/42)		7.1 (6.3)	≥2	9 (7)	
** Social support**^†^			**Health services utilization**		
Low	66 (48)		** Hospital length of stay** (days)		7.1 (2.9)
High	72 (52)		** Discharged directly home**	120 (87)	
			** Post-surgery community physiotherapy** (hours)		14.7 (18.3)

^*^n = 125 – CND $.

^†^ Social support was dichotomized around the median score: Low (≤ 80) and High (>80).

^‡^ Body mass index.

^§^ n = 132.

^∣∣^WOMAC pain score at enrolment on pre-surgery wait list dichotomized into presence or absence of contralateral knee pain.

^¶^In-hospital complications: wound infection (n = 5), implant infection (n = 4), fracture or dislocation (n = 3), knee ankylosis and manipulation (n = 4), cardiovascular/pulmonary/ circulatory complications (n = 12), peripheral/central nervous system involvement (n = 9), urinary infection (n = 3), acute confusion (n = 1), tendon and ligament rupture (n = 1) or blood transfusion (n = 14).

**Table 2:** Overall changes in WOMAC and SF-36 scores of the study participants from before surgery to 6 months after total knee arthroplasty (n = 138)

	**Mean score before TKA**^**† **^**(SD)**	**Mean score 6 months after TKA **^**† **^**(SD)**	**Change in score **^‡^**(SD)**	**95% CI**	***p *value**
**WOMAC**					
** Pain**	44.2 (17.1)	77.3 (17.2)	33.1 (21.8)	29.5 to 36.8	<0.001
** Function**	42.1 (15.5)	71.9 (18.1)	29.8 (20.5)	26.3 to 33.2	<0.001
**SF-36**					
** Physical functioning**	18.5. (15.3)	40.9 (21.7)	22.4 (22.6)	18.6 to 26.2	< 0.001
** Role physical**	36.5 (21.7)	56.6 (25.6)	20.1 (28.9)	15.3 to 25.0	< 0.001
** Bodily pain**	27.6 (10.7)	52.1 (11.2)	24.5 (19.1)	21.3 to 27.7	< 0.001

TKA: Total knee arthroplasty.

SD: standard deviation.

CI: confidence interval.

^**†**^ Scores presented as %. Higher scores always sign a better condition.

^‡^ Positive changes in score sign an improvement of the condition.

Mean SF-36 scores at 6 months after surgery for the three sub-scales were all significantly below the age matched Canadian normative data (p < 0.05), as the mean normal scores for the physical functioning, role-physical and bodily pain scales are respectively: 75.7 (95% CI: 74.9 – 76.5), 76.2 (95% CI: 74.9 – 77.5) and 74.0 (95% CI: 73.1 – 74.8) [50].

### Multivariate regression analyses

Because there were no differences in conclusions whether we used untransformed or rank-transformed dependent variables, the final models were built with untransformed scores. In the final models, all potential confounders were assessed and no other adjustments were necessary.

Results of multivariate analyses on the WOMAC pain and function scores are presented in Table 3. Higher preoperative pain level on the WOMAC pain scale, cruciate retaining implant type and the number of in-hospital complications were significantly associated with higher pain levels at 6 months after TKA and explained 11% of the variance of the WOMAC pain score (multiple correlation coefficient r = 0.33). Higher preoperative functional limitations on the WOMAC function scale, marital status (single, separated, divorced or widowed), being unemployed or retired and the number of in-hospital complications were significantly associated with worse functional limitations at 6 months and explained 16% of the variance of the WOMAC function score (multiple correlation coefficient r = 0.4).

**Table 3:** Association between the study participants’ characteristics and the WOMAC scores 6 months after total knee arthroplasty (n = 138)

**WOMAC SCORE 6 MONTHS AFTER TKA**^**†**^	**β**^‡^	**95% CI**	***p value***
**Pain score (R**^**2**^ **= 0.11)**			
WOMAC pain score before surgery (%)	0.25	0.08 to 0.41	0.004
Cruciate retaining implant^§^	- 8.21	−15.01 to −1.34	0.02
Number of in-hospital complications^¶^	- 5.96	−10.76 to −1.16	0.01
**Function score (R**^**2**^ **= 0.16)**			
WOMAC function score before surgery (%)	0.35	0.16 to 0.54	<0.001
Marital status (single, separated, divorced or widowed) °	- 6.84	−12.74 to −0.95	0.02
Occupational status (unemployed or retired) ^Δ^	- 7.77	−14.70 to −0.87	0.03
Number of in-hospital complications^¶^	- 5.04	−9.83 to −0.26	0.04

TKA: Total knee arthroplasty.

^**†**^ Stepwise multiple regression analysis.

^‡^ Multivariate unstandardized linear regression coefficients. For each unit of the participants’ characteristics, there is in average a β increase (+) or a decrease (−) on the WOMAC score at 6 months after TKA. A positive β has a positive effect on the participants’ condition and a negative β has a negative effect.

WOMAC pain and function scores before surgery treated as continuous variables. Higher pain or function scores signify better patient status.

Other independent variables are categorical.

^§^ Cruciate retaining compared to postero-stabilized implant.

^¶^ Reference category = 0 complication compared to 1 and ≥ 2 complications.

° Single, separated, divorced or widowed compared to married or common law status.

^Δ^Unemployed or retired compared to employed.

Results of multivariate analyses on the SF-36 scores are presented in Table 4. Lower preoperative HRQoL on the SF-36 physical functioning scale, presence of contralateral knee pain before surgery, higher preoperative psychological distress and the burden of comorbidities measured by the CIRS were significantly associated with worse HRQoL 6 months after TKA and explained 23% of the variance of the SF-36 HRQoL physical functioning score (multiple correlation coefficient r = 0.48).

**Table 4:** Association between the study participants’ characteristics and the SF-36 health-related quality of life scores 6 months after total knee arthroplasty (n = 138)

**SF-36 SCORE 6 MONTHS AFTER TKA **^**†**^	**β**^‡^	**95% CI**	***P value***
**Physical functioning (R**^**2**^ **= 0.23)**			
SF-36 Physical functioning score before surgery (%)	0.24	0.01 to 0.47	0.036
Presence of contralateral knee pain before surgery^§^	- 12.68	- 20.37 to - 4.99	0.001
Psychological distress (PSI score /42)	- 0.54	- 1.06 to - 0.02	0.04
Comorbidities (CIRS score / 56)	- 2.60	−4.11 to - 1.08	<0.001
**Role-physical (R**^**2**^ **= 0.17)**			
SF-36 Role-physical score before surgery (%)	0.31	0.12 to 0.51	0.01
Comorbidities (CIRS score /56)	- 2.02	- 3.91 to - 0.13	0.04
Number of in-hospital complications^¶^	−7.41	- 14.23 to - 0.69	0.03
**Bodily Pain (R**^**2**^ **= 0.13)**			
SF-36 Bodily pain score before surgery (%)	0.72	0.43 to 1.01	<0.001
Presence of contralateral knee pain before surgery	−7.45	−14.34 to - 0.55	0.03
Comorbidities (CIRS score /56)	- 1.97	- 3.37 to - 0.57	0.001
Number of in-hospital complications^¶^	−5.47	- 10.52 to - 0.39	0.04

TKA: Total knee arthroplasty.

^†^ Stepwise multiple regression analysis.

^‡^ Multivariate unstandardized linear regression coefficients. For each unit of the participants’ characteristics there is on average a β increase (+) or a decrease (−) on the SF-36 score. A positive β has a positive effect on the participants’ condition and a negative β has a negative effect.

SF-36 scores before surgery, psychological distress and comorbidities treated as continuous variables. Higher scores signify better patient status.

Other independent variables are categorical.

^§^ Presence compared to absence of contralateral knee pain as measured by the WOMAC pain scale.

^¶^ Reference category = 0 compared to 1 or ≥ 2 complications.

Lower preoperative HRQoL on the SF-36 role-physical scale, the burden of comorbidities measured by the CIRS and the number of in-hospital complications were significantly associated with worse HRQoL 6 months after TKA and explained 17% of the variance of the SF-36 HRQoL role-physical score (multiple correlation coefficient r = 0.41).

Lower preoperative HRQoL on the SF-36 bodily pain scale, presence of contralateral knee pain before surgery, the burden of comorbidities measured by the CIRS and the number of in-hospital complications were significantly associated with worse HRQoL 6 months after TKA and explained 13% of the variance of the SF-36 HRQoL bodily pain score (multiple correlation coefficient r = 0.36).

## Discussion

In this prospective cohort study, 138 participants were recruited and followed up 6 months after TKA to measure pain, functional limitations and HRQoL and to identify preoperative and perioperative determinants associated with these outcomes. Several variables were found to be significantly associated with worse outcomes 6 months after TKA. The identification of these determinants could help identify patients at risk of poorer outcome and help manage these patients more efficiently.

Participants showed a significant improvement 6 months after TKA in terms of pain, functional limitations and HRQoL. Several determinants of 6-month TKA outcomes were identified in this study, with multivariate models of the WOMAC and SF-36 scales explaining variances ranging from 11% to 23%. Although for some outcomes (e.g. WOMAC pain score) the explained variance was small, these results are in accordance with those of other studies that used the WOMAC and SF-36 to model determinants of post-surgery outcomes [10,12,13,16]. It is important to point out that the magnitude of the associations found in this study, if taken separately, may not be clinically important, but if more than one of these characteristics is present in a patient, they are likely to have a clinically important impact [49].

Pre-operative status in terms of pain, functional limitations and HRQoL was significantly associated with post-operative status and these associations were consistent across the 2 scales of the WOMAC and the 3 scales of the SF-36 used in this study. Other authors have identified that pre-operative status is associated with post-operative status, and the strength of the associations we found is very similar to the results presented in other studies regarding TKA patients [10,11,13,15,16,51].

Although there is abundant literature on the predictors of complications such as cardio-vascular events, embolisms, thrombophlebitis, wound infection or blood transfusions following TKA, there are relatively few studies that have looked at the association of surgical and perioperative complications with the outcomes of TKA as we did. We found that the number of in-hospital complications was significantly associated with post-operative outcomes 6 months after TKA in terms of pain, functional limitations and HRQoL. This determinant was found significant for the pain and the function scales of the WOMAC as well as for the role-physical and bodily pain scales of the SF-36, but was not significant for the physical functioning scale of the SF-36 (p = 0.09). Two other studies, which measured TKA complications, but after hospital discharge, found this determinant significantly associated with the WOMAC scores after surgery [12,52]. In those studies, patients who suffered more complications after discharge were found to have more pain and more functional limitations; in one study these findings were at twelve months after TKA and focused on obese patients [12] and in the other study, at a seven year follow-up for a general TKA patients population [12]. Only one other study in the literature did not find a significant association between the number of in-hospital complications and functional limitation six months after TKA [10].

The burden of comorbidities measured with the CIRS tool was significantly associated with worse HRQoL for the three SF-36 scales 6 months after TKA. The strength of the association was fairly consistent across these 3 scales, as shown by the unstandardized linear regression coefficients ranging from −1.97 to −2.60, even though the confidence intervals were large. Interestingly, this determinant was not found to be significant in the WOMAC models. Notwithstanding that physical functioning, role physical and bodily pain scales are responsive to physical limitations related to knee disorders, TKA and post-surgery recovery, one potential explanation for this finding is that the SF-36 questionnaire, a general HRQoL questionnaire, does not focus only on the involved knee contrary to the WOMAC questionnaire [36].

The presence of contralateral knee pain before surgery was found to be a significant determinant of post-surgery HRQoL SF-36 physical functioning and bodily pain. This represents an interesting finding confirming again the negative impact of bilateral knee pain on the outcomes of TKA. These results are in line with previous published results for this cohort of patients, where participants with contralateral knee pain at inclusion on pre-surgery TKA wait lists had worse pain, functional limitations and HRQoL [22,23]. Only Jones et al. had previously looked at that possible association in patients undergoing total hip arthroplasty. They found that contralateral involvement was only a significant determinant for function 6 months following surgery [19].

It is interesting to note that participants who received a cruciate retaining implant had significantly more pain 6 months after TKA than patients who received a postero-stabilized implant, although the magnitude of the difference found may not be considered clinically important. A similar trend was seen in functional limitations measured by the WOMAC function scale, but it was not statistically significant for this model (p = 0.08). There is no consensus among surgeons whether to use a cruciate ligament retaining design or a posterior-stabilized design for TKA. However, the surgical technique for cruciate retention is technically more challenging especially if the posterior cruciate ligament requires balancing, as it may lead to pain and instability of the reconstructed knee [53]. Clearly, more studies are needed to fully conclude on which design yields the best results, since many studies conducted on this topic presented serious methodological problems and were underpowered [53]. These studies often used intermediary impairment measures such as knee range of motion (instead of measure of functional performance), or they used non-validated outcome measures with poor psychometric properties.

Psychological distress level was low in this cohort of patients. Nonetheless, it was significantly associated with worse HRQoL physical functioning scores. Other studies have outlined the important role of psychological distress on the health status of patients suffering from knee pain or undergoing TKA [8,54,55]. In our study, subjects married or living in common-law had a better function compared to single, separated, divorced or widowed subjects. Further adjustment of this regression model with social support did not change the strength of the association between marital status and the WOMAC function score. Therefore, we believe that this association is more likely related to the help of the spouse on coping skills than to an effect of social support [56]. It remains unclear why being employed was a significant independent determinant of better functional status 6 months after TKA. Further adjustment of this model for age did not change the strength of the associations, nor the number of post-surgery physiotherapy hours, and the final model is adjusted for pre-operative functional status, three important potential confounding factors. We hypothesize that workers may suffer from a response shift as a proportion of them may have been returning to work after their surgery. Therefore, the perceived ability of the newly reintegrated workers to be able again to accomplish their work may bring them to rate their function more favorably compared to the non-workers. However this should have reflected on the SF-36 models, an effect that was not observed.

### Strengths and limitations of the study

Strengths of this prospective cohort study include an initial high participation (81.8%) and follow-up (90.1%) proportions. There is no indication of a selection bias, as there were no significant differences between participants and eligible non-participants on age and gender and no significant differences in terms of pain, functional limitations and HRQoL before surgery between participants who did and those who did not complete the 6-month interview (data not shown). Also, orthopaedic surgeons acting as treating physicians during the study could not influence the patients’ participation as they had not been informed of the study start and stop dates. Several pre- and perioperative factors were significantly associated with worse pain, functional limitations or poorer HRQoL at follow-up. One of the strengths of our study is that many of these factors have a consistent effect across the scales of the WOMAC or the SF-36, which further supports the validity of our results. Other strengths include thorough and relevant independent variables selection. Further adjustments of the regression models with other potential confounding factors (presented in Table 1) only marginally changed the strength of the associations and were therefore not kept in the final models.

Although our study had adequate statistical power, the precision of some estimates was low, as shown by the large confidence intervals found in different models. A larger cohort of participants would have allowed for a more precise estimation of the strength of the associations found here.

We used the total number of in-hospital complications and did not take into account the severity of the complications. To our knowledge, there is no validated tool that exists to assess the severity of complications for that population and we therefore resolved to use only the number of complications in the analyses. Another limitation was that the main outcome measures were self-reported and performance-based measures were not included. The WOMAC and the SF-36 have been found to be valid instruments; still, it has been reported that performance-based measures provide distinct impressions of pain and function that complement self-reported measures [57]. Therefore, the associations or strength of associations between patients’ characteristics and performance-based measures could be different from the findings of our study. It is important to point out that this study focused on patients scheduled for primary unilateral knee replacement and excluded patients undergoing a revision or with a previously implanted contralateral knee replacement or a hip replacement, therefore results may differ for these patients.

## Conclusions

Several preoperative and perioperative variables are associated with pain, functional limitations and HRQoL 6 months after TKA, namely baseline levels of pain, functional limitations and HRQoL, type of implant, occupational and marital statuses, number of in-hospital complications, contralateral knee pain, psychological distress and comorbidities. The magnitude of these associations taken separately may not be clinically important but if more than one of these characteristics is present in a patient, they are likely to have a clinically important impact. The patient’s characteristics found in this study could help identify patient who are at risk of complications or may have a poorer outcome following surgery. These results will contribute to manage TKA patients more efficiently. These results will also help in the design of clinical trials aimed at the evaluation of interventions to optimize management of TKA patients at higher risk of poor outcome.

## Competing interests

The authors declare that they have no competing interests.

## Authors’ contributions

FD participated in the design, coordination and collection of data. He performed the statistical analysis and drafted the manuscript. CED participated in the design, coordination and helped drafted the manuscript. ELB participated in the design, coordination and helped drafted the manuscript. RB participated in the design and helped drafted the manuscript. FC participated in the design and helped drafted the manuscript. PF participated in the collection of data and helped draft the manuscript. All authors read and approved the final manuscript.

## Pre-publication history

The pre-publication history for this paper can be accessed here:

http://www.biomedcentral.com/2052-1847/5/2/prepub
